# Low cycle fatigue of thin-wall printed Onyx in energy absorption

**DOI:** 10.1016/j.heliyon.2025.e42120

**Published:** 2025-01-21

**Authors:** Moises Jimenez-Martinez, Guillermo Narvaez, Paulina Diaz-Montiel

**Affiliations:** a Tecnologico de Monterrey, Escuela de Ingeniería y Ciencias, Via Atlixcayotl 5718, Col. Reserva Territorial Atlixcayotl, C.P. 72453 Puebla, Mexico; b Volkswagen de México, Technical development México, Puebla, 72730, Mexico; c University of San Diego, 5998 Alcalá Park, San Diego, CA 92110, USA

**Keywords:** Fatigue, Energy absorption, Peak crushing force, Onyx, Additive manufacturing

## Abstract

Passive safety systems have been evaluated for their ability to transform impact energy into deformation to reduce the probability of damage to passengers during crash events. Low-speed impacts are common during collisions and many structural components are not replaced after such collisions because of the recovery of visual components such as the bumper fascia. However, automotive foams and brackets deform permanently in case a new impact fails to dissipate energy. In this study, a thin-walled printed Onyx component was fabricated via additive manufacturing. This material was used to dissipate energy at low-cycle fatigue and recovery in the peak crushing force after the first crushing cycle. The thin-wall crash box printed with Onyx, can be designed to recover energy absorption in different regions of the crushing displacement. The first peak crushing force and the mean crushing force are recovered according to the geometry and small displacements. However, in medium and long crushing displacements, at the end of the compression its dissipation capacity is increased. Onyx printed mechanical absorber withstand fifteen load cycles, recovering the peak load 19.25%.

## 1 Introduction

Active and passive safety systems are used in automotive structures. Active safety systems prevent accidents and improve manoeuvrability using advanced driver assistance systems comprising sensors and algorithms by enhancing active safety due to improvements in lane-change systems, side assist blind-spot warning and forward collision warning [1]. Passive safety systems prevent collision from the moment an accident occurs until the automobile stops, dissipating kinetic energy. Safety devices such as seat belts, airbags and other automotive body structures protect passengers and pedestrians from biomechanical damage [2]. The probability of such damage can be reduced by implementing a crashworthiness design, which is the ability of a structure to protect its contents such as occupants, pedestrians and cargo during an impact [3], [4]. The crashworthiness is evaluated for different vehicles such as aircraft, automobiles and subway vehicles [5], [6]. Based on the type of impact different parts of a vehicle work as mechanical attenuators to reduce the transmission of kinetic energy and its inertial effects [7], [8]. Vehicle collisions have different impact stages. The first impact occurs when vehicle impacts another vehicle moving at different velocities or one of them is in repose. The forces are then transferred to the occupants wearing seatbelts and in contact with airbags. The final impact occurs when the passenger moves and their internal organs are impacted [9] due to the amplitude of load (acceleration) and intensity [10]. The acceleration and deceleration are determined with a reference of gravity [11]. The components in the front end assembly of cars deal with kinetic energy such as the crash box, which is a thin-walled component with geometries as square, rectangle, hexagon, circular, octagon, top-hat, beam and tapered beam sections [12], [13], [14], [15]. Crash boxes are fabricated via stamping and have a stiffener or corrugated designs to manage deformation and energy dissipation, thereby preventing the vehicle's main structure from any major damage. These structures dissipate and control the energy via progressive folding, wherein kinetic energy is transformed into deformation energy [16]. Crash box materials typically include aluminum and steel [17]. However, the use of thermoplastic fibre-metal laminates for crash boxes has also been proposed [18].

Herein, thin-walled printed Onyx is proposed owing to its viscoelastic behaviour that recovers accumulated damage in low-cycle fatigue under crushing loads. Crushing tests were performed to elucidate if the component could withstand the impact of a low-cycle crushing load. Thus, thin-walled Onyx structures can be used as deformation elements to primarily recover from peak crushing force (PCF) in low-cycle fatigue and mean crushing force response under crushing loads. Different geometries were evaluated to define their influence on the recovery process, along the compression load. This allowed to analyze the first and last peak of crushing load, as well as the average value. The use of thin wall elements printed from onyx, allows to develop novel crash boxes by allowing energy recovery.

## 2 Crashworthiness

All vehicles have protection elements—either part of the assembly or in an external device—that prevent their main structure from damage. In trains, the cowcatcher is located in the front end and it primarily removes obstacles on tracks. However, in case of collisions, it first absorbs energy and prevents the main structure from damage [19], [20].

**Figure 1:**
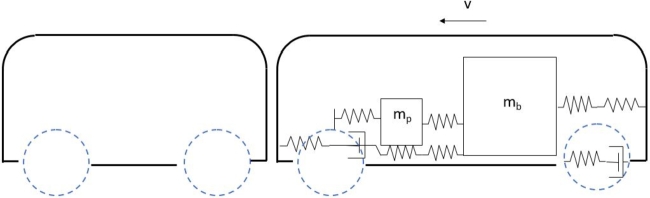
Schematic of a viscoelastic model of a vehicle crash.

Fig. 1 shows the impact between two vehicles; deceleration occurs if both vehicles are moving or acceleration occurs if either of the vehicle is in repose. In such cases, a typical car body is split into two or three boxes and the last box is in vehicles with trunk. One box is for the compartment and the other one is for the frontal part where the engine is normally located. This frontal box contains an energy absorber that works as a mechanical fuse to prevent critical physical damage on other components. The energy is not transmitted if the components collapse, thereby reducing time and budget for repair.

**Figure 2:**
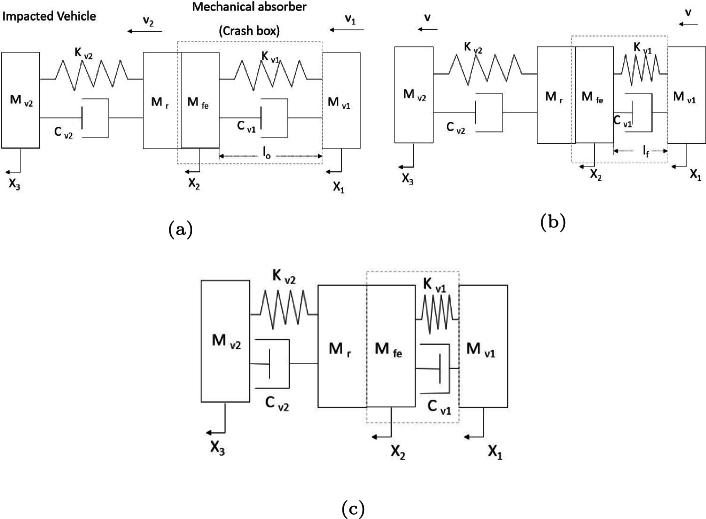
Analysis of energy absorption during impact: (a) at the beginning of the impact, (b) during the impact and (c) after the impact.

Fig. 2 shows different impact stages. $m_{v1}$ $k_{v1}$ and $c_{v1}$ are the concentrated mass, stiffness and damping of the vehicle that causes impact, respectively and $m_{v2}$ $k_{v2}$ and $c_{v2}$ are the concentrated mass, stiffness and damping of the impacted vehicle. $M_{fe}$ is the concentrated mass of the front end and Mr is the mass of the rear box (rear part) of the vehicle impacted. Before the impact, both vehicles are in movement or one of them is in repose (Fig. 2a). When the impact begins, both inertia loads are coupled (Fig. 2b). The vehicles change the kinetic energy into deformation energy using physical properties. After the impact, the element is deformed (Fig. 2c).

**Figure 3:**
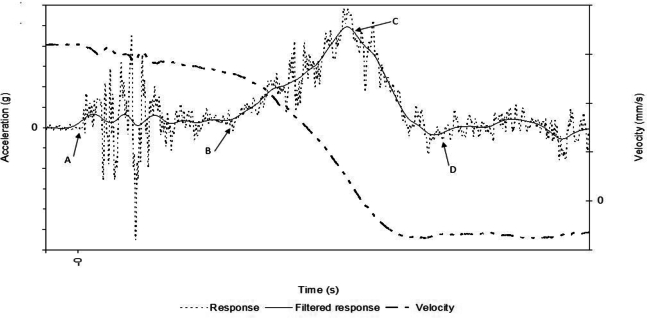
Time history of a crash event.

Fig. 3 shows the time history of an impact. The impact starts at point *A* 2b known as the trigger. As the inertia transitions to couple the movement of both vehicles (from point *A* to point *B*), the structures collapse until it reaches maximum deceleration (point *C*). Here, the velocity can rebound after reaching the highest deceleration peak and at point D, the major contribution of the energy dissipation ends. The response signal denotes the raw deceleration data and the filtered response is the acceleration with a low-pass band filter of 100 Hz. Above this sampling rate, the signal is evaluated as noise in structural analysis.

**Figure 4:**
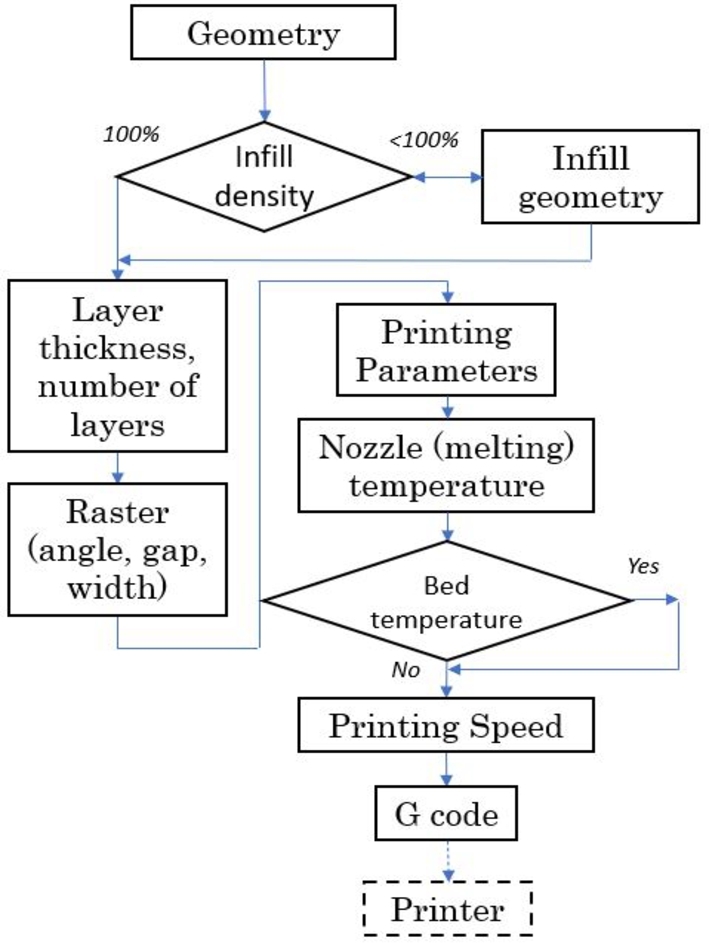
Printing parameters.

Based on the material of the vehicle body, the energy absorption devices are manufactured using aluminium, steel or composites materials using traditional manufacturing processes such as stamping and additive manufacturing (AM) [21], [22], [23], [24]. AM yields versatile designs and components can be manufactured without tooling. Fig. 4 shows the component manufacturing process—from the geometry to the code used for the printer to generate the components.

In *AM*, many parameters have to be defined, which impact the physical characteristics of manufactured components such as surface finish and their mechanical properties. The mechanical strength of components is impacted by parameters such as the infill, layer thickness, nozzle and bed temperature, position on the bed (raster) and printing speed, which are considered by the slicer to generate a *G* code and submitted to the printer.

## 3 Energy absorption

**Figure 5:**
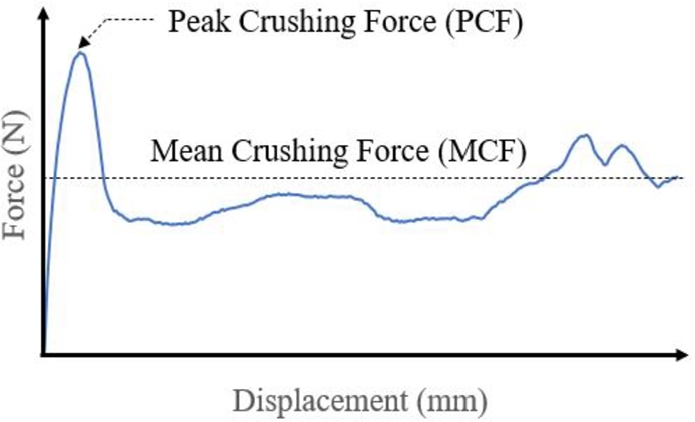
Crashworthiness indicators.

The crushing performance of thin-walled structures depends on their characteristics, such as strength, design characteristics (dimensions) and materials used for their fabrication. These characteristics are modified to control the crushing behaviour to manage the crashworthiness of the structures using energy absorption indicators. The amount of energy dissipated as deformation is determined using absorption indicators that measure the crushing force and displacement (Fig. 5).

Energy absorption can be managed using different strategies such as by installing mechanical absorbers to absorb the Peak Crushing Force (PCF) resulting from the collision of vehicles moving with different velocities due to the load that causes displacement. Such absorbers are based on the mechanical properties and the crash box design. The Specific Energy Absorption (SEA) is determined based on the Energy Absorbed (EA) by a structure during a crush per total mass of the structure (Eq. (1)):

(1) $$SEA=\frac{EA}{m}=\frac{\int_{o}^{\delta_{c}}F(\delta )d\delta }{m}$$

where $\delta_{c}$ is the maximum displacement of a rigid box collapsing the structure, *m* is the total mass and F(δ) is the variation in force as a function of displacement *δ*.

The mean crushing force (MCF) is the ratio of energy absorbed to length reduction (*δ*), as shown in Eq. (2):

(2) $$MCF=\frac{EA}{\delta }$$

The relation between the inverse of the *PCF* multiplied by the *MCF* is defined by the Crushing Force Efficiency (CFE) as follows:

(3) $$CFE=\frac{MCF}{PCF}$$

The optimal energy dissipation performance of the crash box can be determined by combining these indicators [4].

## 4 Materials

Printed Onyx is a composite material composed of nylon polymer (Polyamide 6) reinforced with 10%-20% chopped carbon fibres [25], [26]. It has high elasticity owing to the presence of flexible-chain polymers and can recover from large displacements. This composite material experiences creep under permanent loads. When used with metals, the temperatures should be more than 50% the melting point of Onyx, as below this temperature, the material is not entirely elastic. This change is caused by dislocation in grain size. Polymers exhibit a different creep behaviour than printed materials at all temperatures. The forces induce molecular motions in the polymer, generating relaxation and dissipation energies, to recover the mechanical strength after crushing load based on the elastic (Eq. (4)) and viscous behaviours (Eq. (5)).

(4) $$\sigma v_{1}=\xi \cdot \varepsilon_{1}$$

(5) $$\sigma v_{1}=\eta \cdot {\overset{˙}{\varepsilon }}_{2}$$

where *ξ* is a constant; $\sigma v_{1}$ and $\sigma v_{2}$ are the stresses for the elastic and viscous behaviour, respectively; ${\overset{˙}{\varepsilon }}_{2}$ is the strain rate.

Assuming that $\sigma =\sigma v_{1}=\sigma v_{2}$, the total strain is $\varepsilon =\varepsilon v_{1}+\varepsilon v_{2}$; the stress is included in the total strain.

(6) $$\overset{˙}{\varepsilon }=\frac{1}{\xi }\overset{˙}{\sigma }+\frac{1}{\eta }\sigma$$

Using a load ramp constant, the stress $\sigma_{o}$ is constant and expressed as follows:

(7) $$\overset{˙}{\varepsilon }=\frac{1}{\eta }\cdot \sigma$$

The strain as a function of time is

(8) $$\varepsilon (t)=\frac{\sigma_{o}}{\xi }+\frac{\sigma_{o}}{\eta }$$

From Eqs. (8) and (9), the creep modulus is defined as

(9) $$\varepsilon (t)=\frac{\sigma_{o}}{\xi }+\frac{\sigma_{o}}{\eta }$$

The expected behaviour depends on the material characteristics and strain at different times.

## 5 Additive manufacturing

Three-dimensional (3D) printed samples are widely characterised to determine their structural integrity. *AM* reduces the time required for component design to its final manufacturing, as it does not require the design and production of specialised tools. Thus, geometries can be created cost-effectively, which cannot be obtained using traditional manufacturing methods. *AM* is highly profitable in the production of short series or prototypes, particularly when dealing with complex and organic geometries. In fused deposition modelling (FDM), polymers are heated above their glass transition temperatures and deposited layer by layer using a nozzle. However, the composites manufactured using *FDM* have high surface roughness levels and poor mechanical performance than those fabricated using traditional manufacturing methods.

**Figure 6:**
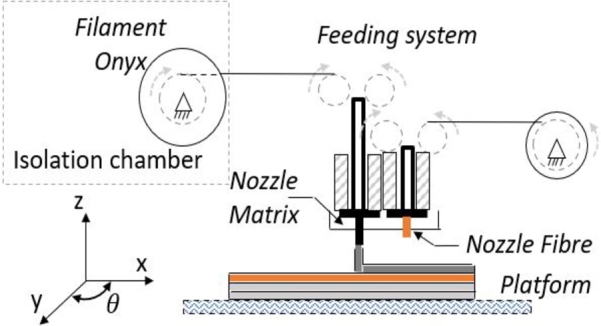
Schematic printing process.

All specimens in this study were printed using the Markforged Mark Two printer and an Onyx black filament of 1.75-mm diameter. The printing parameters were a nozzle temperature of 273 °C, a raster of 45° [27] and a layer thickness of 0.1 mm owing to the high compressive strength of specimens [28]. Fig. 6 shows the printer containing an isolation chamber due to the sensitivity of the filament to humidity.

## 6 Results and discussion

**Figure 7:**
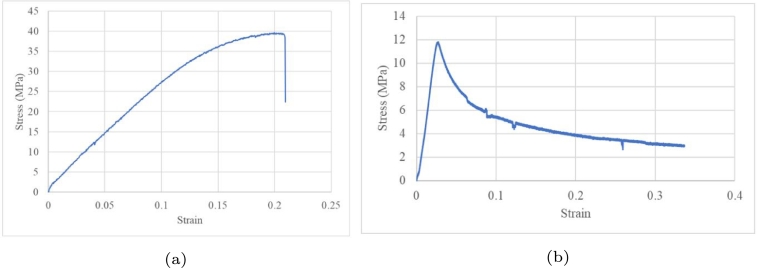
Quasistatic tests conducted at a rate of 1 mm/min under (a) tensile and (b) compression loads.

A dog bone composite was evaluated under a tensile load (Fig. 7a) and compression load (Fig. 7b). The ultimate tensile strengths were 39.6 and 11.8M Pa for tensile and compression tests, respectively. During compression, the structural behaviour of the composite shows a smooth transition until failure.

**Figure 8:**
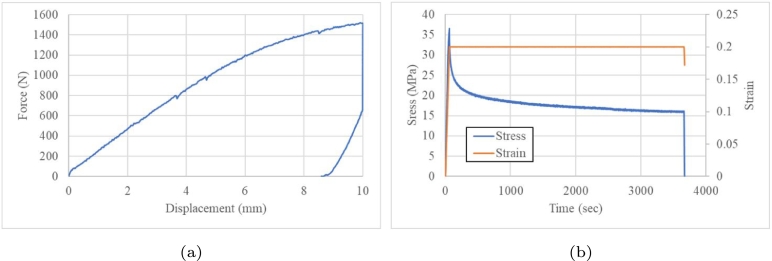
Stress relaxation test at 10 mm: (a) force vs displacement and (b) stress relaxation tests.

Quasistatic tests with ramps at load and displacement were performed. The creep curve shows the elastic instantaneous response and retarded elastic deformation due to the viscous behaviour of the composite. Although this deformation is partially reversible, it is time-consuming, which impacts the energy absorption. These tests were performed to determine the recovery behaviour under different load profiles and recovery times. A displacement ramp at 10 mm was applied for 3,600 s and the peak load was 1,521.4 N. The component experienced a catastrophic failure when the ramp went back to 0 mm, breaking it during unloading. See Fig. 8.

**Figure 9:**
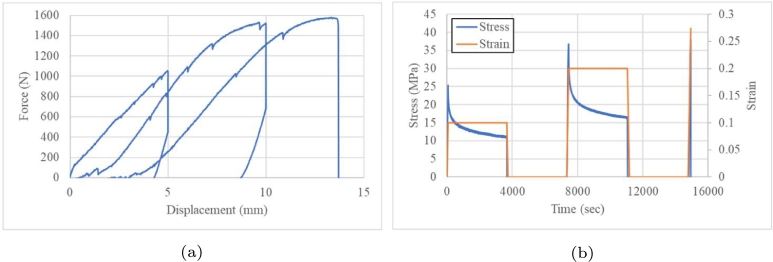
Incremental load every 1,000 N: (a) Force vs displacement and (b) load as a function of time.

To analyse the component response at different strains, the load was split every 5 mm (Fig. 9a). Hardening occurred due to failure at the beginning of the third cyclic load, at 1,590 N. Under this displacement, the load was reduced to 0 N (Fig. 9b).

**Figure 10:**
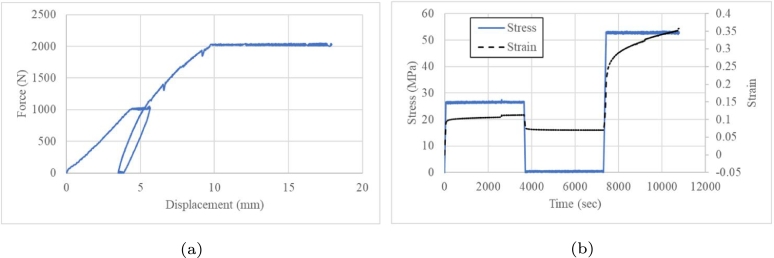
Incremental load of 500 N (a) cyclic load and (b) creep test.

Fig. 10 shows that the component lost its stiffness when a 1000-N load was applied for 1 h and the displacement was increased from 4.27 to 5.26 mm. To reach 0 N, the component moved from 3.82 to 33.5 mm before the load ramp. As the load reached 2000 N in 3,600 s, the component was broken as the unloading began and the displacement increased (Fig. 9a).

**Figure 11:**
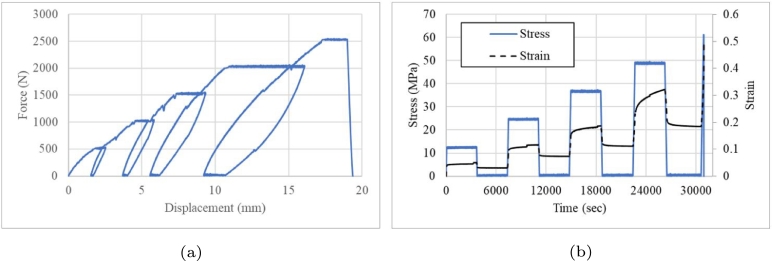
Incremental load of 500 N (a) Force vs displacement and (b) loads as a time function.

Instead of 1,000 N, incremental ramps were applied every 500 N. Interestingly, component failure was observed at 2,500 N instead of 2000 N. Fig. 10b shows the increasing displacement in response to the load. This trend in the fourth cycle is similar to that in the second load cycle (Fig. 11b). The difference is that the load of 2000 N was the same but the displacement decreased. The first one reaching a failure and the second one recovering with a sudden failure at 2,500 N load level. This shows a hardening behaviour, similar to previous tests (Fig. 10b).

**Figure 12:**
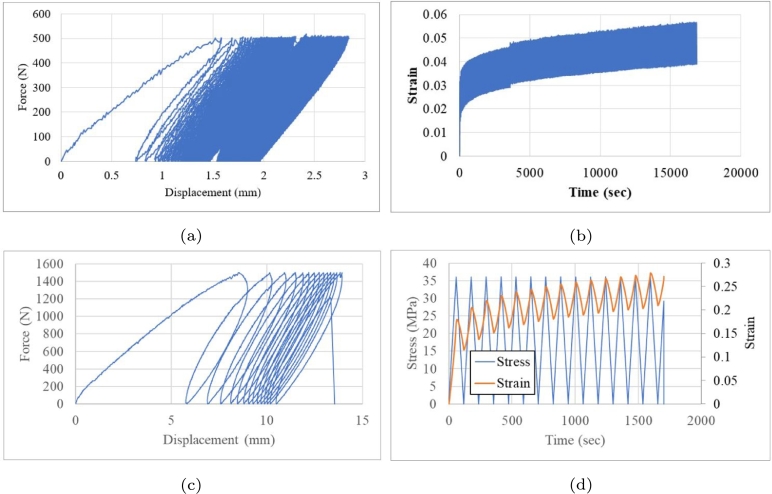
Cyclic tests with controlled loads: (a) maximum load of 500 N (b) cyclic strain, (c) maximum load of 1,500 N and (d) stress relaxation test.

The cyclic behaviour of component was evaluated to analyse its dynamic response under and accumulated damage. Figs. 12a and 12b show the response under minimum and maximum loads of 1 and 500 N, respectively. The test was suspended (run out) after 16,200 s. Then, a new component was evaluated with a maximum load of 1,500 N (Fig. 12c). Fig. 12d shows the stress relaxation, wherein similar displacement behaviour was observed at lower loads. However, the component broken during the fifteenth cycle without completing the cyclic load.

**Figure 13:**
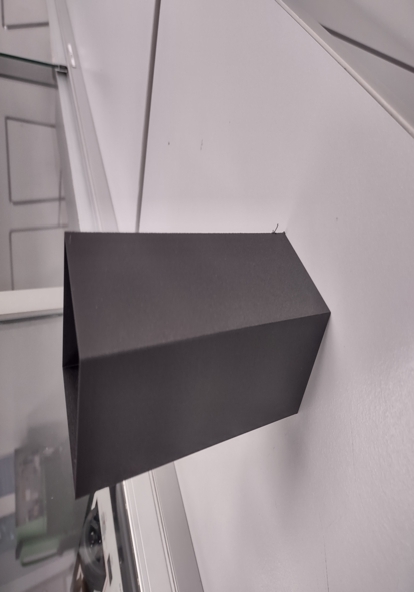
Crash box printed with Onyx.

A crash box was fabricated using Onyx with a rectangular geometry of 50.8 mm, length of 152.4 mm and thickness of 0.8 mm (Fig. 13) to evaluate its mechanical properties.

**Figure 14:**
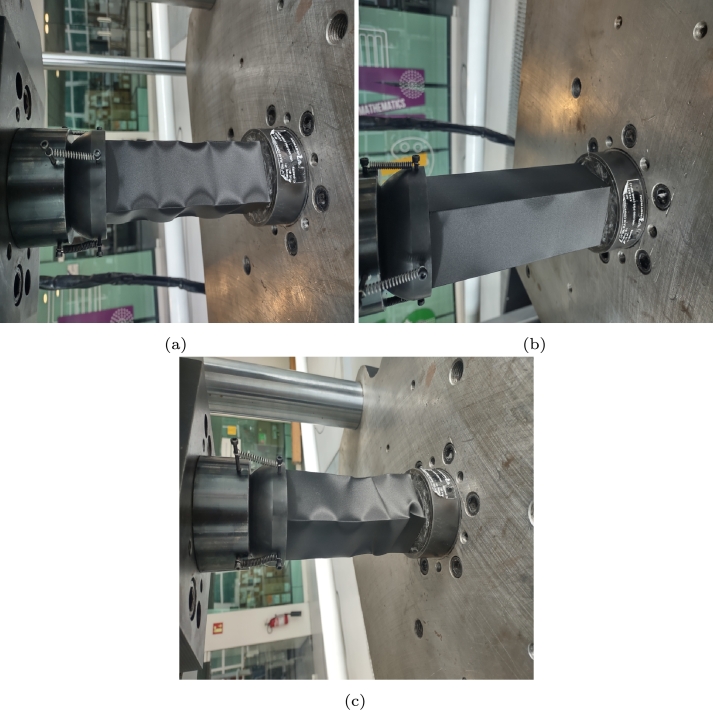
Compression at 1 mm/min, (a) displacement of 8 mm, (b) recovery after the first load and (c) second cycle at 16 mm.

**Figure 15:**
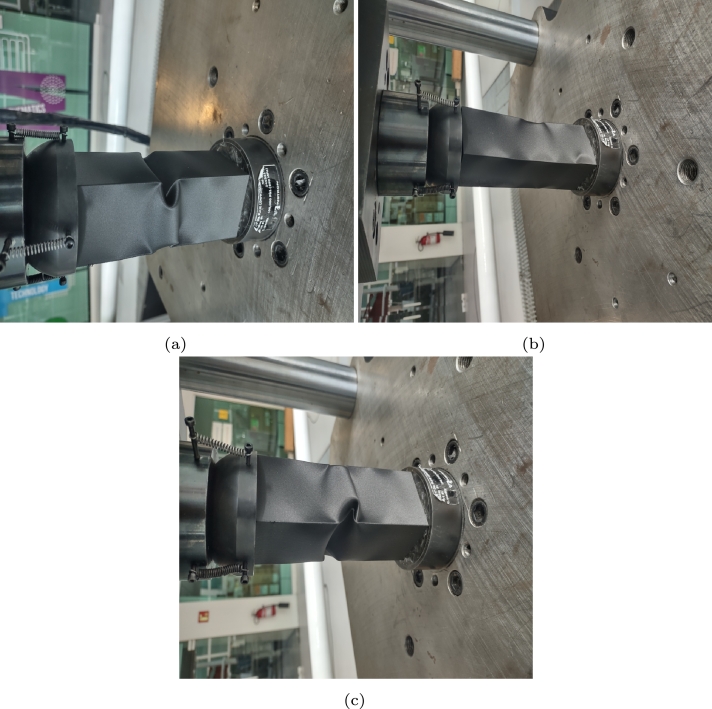
Compression at 70 mm/min, (a) displacement of 8 mm, (b) recovery after the first load and (c) second cycle at 16 mm.

Figure 14, Figure 15 show the mechanical response of the component after applying compression at rates of 1 and 70 mm/min, respectively. In the first test, a displacement of 8 mm was applied, immediately followed by 16 mm. After fifteen days of recovery, 50 mm of displacement was applied.

**Figure 16:**
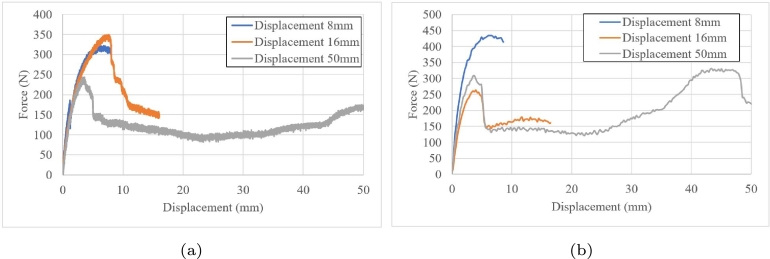
Compression cyclic loads: (a) 1 mm/min and (b)70 mm/min.

Figs. 16a and 16b show the results of compression at rates of 1 and 70 mm/min, respectively. At low displacement rates, the first and second response were similar; however, at 70 mm/min, the second and third cycle response were similar until 5 mm of displacement. These results were somewhat counterintuitive. The last cycle was similar in both rates, not in amplitude nor in second peak value, but it was a similar tendency.

**Figure 17:**
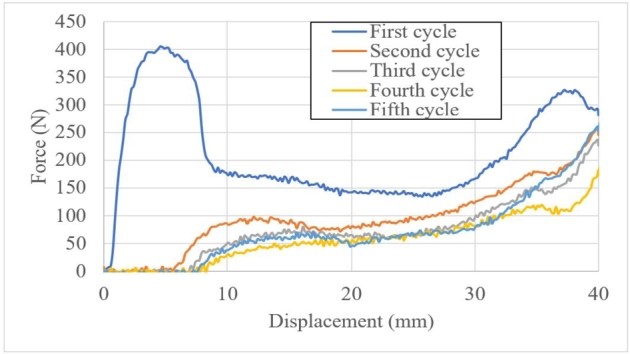
Cyclic loads for the same specimen.

Based on the feasibility of the cyclic load, 5 cyclic ramps were applied until 40-mm displacement and unloaded; results are shown in Fig. 17. At 25% of displacement in length, a second crushing peak was observed and the load was measured. At the beginning of the test, a permanent deformation was observed from 0 to 8 mm. As no load was subjected from the crash box, a second *PCF* was observed at 38.1 mm.

Table 1 shows the normalised *PCF* after applying cyclic loads. The material could recover its length and mechanical strength. The load was applied without any unload time (continuous load and unload cyclic loads) to reach a relative *PCF* for recovery. The second *PCF* reached 24.2% of the first *PCF*, the third *PCF* reached 81.8% of the second PCF and the fourth cycle reached 88.4% of the previous *PCF*, which was the maximum value in the last cycle (84.4%) due to hardening of the composite. Although the last cyclic load cycle had more *PCF* peaks, they were based on the folding process and not directly on the component recovery. The first peak was observed as a response to the strength of the crash box.

**Table 1:** Peak crushing force after cyclic loading.

Cycle load	Force (N)	%PCF	Displacement at initial load (mm)
1	406	100	0
2	98.4	24.24	5
3	80.5	81.81	6.75
4	71.2	88.45	7.5
5	60.1	84.41	8.25

Fatigue life assessment can be performed using degradation models (strength, modulus and stiffness) and damage models [29], [30]. To evaluate the material damage at low-cycle fatigue, the accumulated damage was determined using the loss stiffness and degradation strength model. The material stiffness was determined based on the Young's modulus of composites and components printed via *AM* and *FDM* [31], [32]. The degradation model for fatigue life assessment was developed based on the *PCF*. The relation between the initial value, $PCF_{o}$, the critical *PCF* at failure $PCF_{f}$ and *PCF* at the $n_{th}$ cycle is expressed in Eq (10).

(10) $$D_{n}=\frac{PCF_{o}-PCF_{n}}{PCF_{o}-PCF_{f}}$$

**Figure 18:**
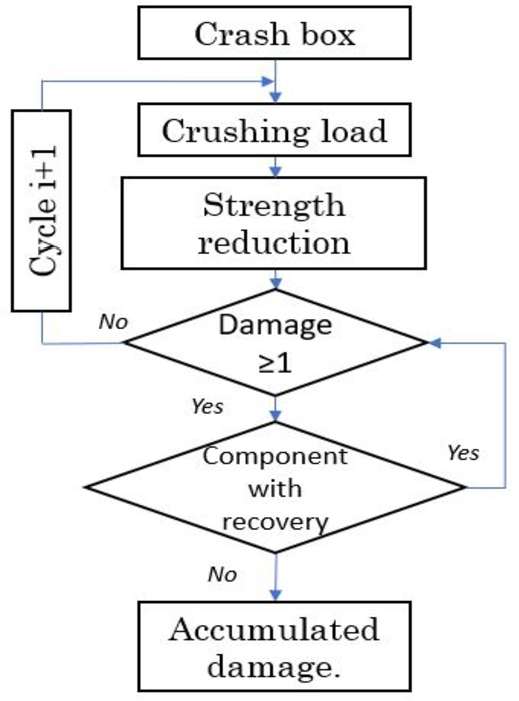
Flow chart of cyclic damage assessment by crushing.

Fig. 18 shows the process of fatigue life assessment of the crash box. The strength was determined at every cycle until the peak crushing load reached a critical value to induce damage; this is denoted as $PCF_{f}$.

**Figure 19:**
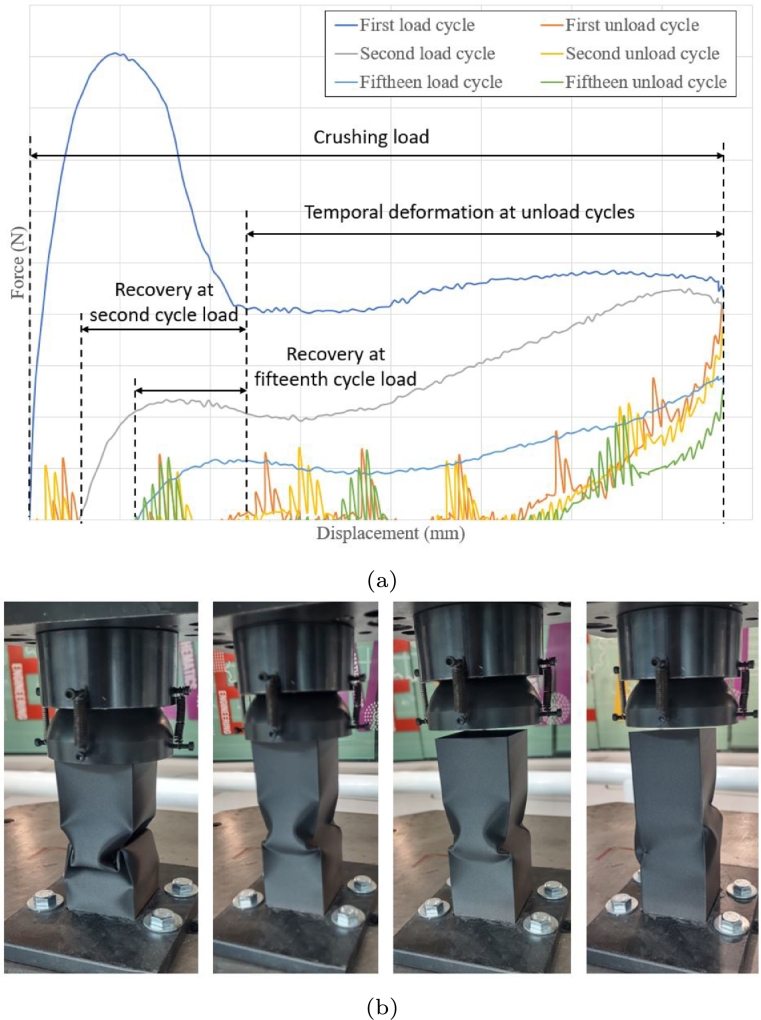
Recovery process after cyclic load, (a) crushing force history and (b) physical damage on crash box.

Fig. 19a shows the acquired response history of force vs. displacement. Fig. 19b shows from left to right the loading process, unloading process of the first cycle, temporary permanent deformation and finally the deformation after fifteen loading cycles.

**Figure 20:**
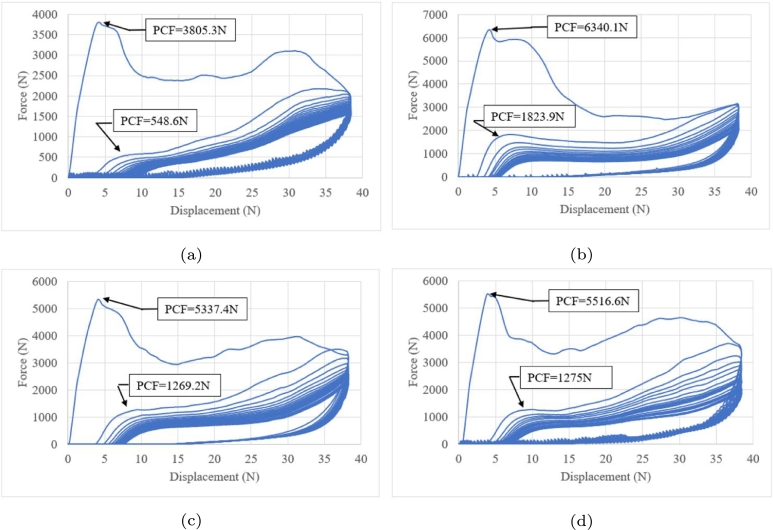
Cyclic behaviour under (a) 1 mm of unload, (b) 30 min of unload, (c) 60 min of unload and (d) 200 min of unload.

A new set of physical tests were performed at different unloading times to analyse the effect of recovery time. The component was fastened with a clevis fastener to prevent its movement. Fig. 20 shows the 25% displacement load until 38.1 mm. Within 1 minute of unloading, the recover load was 14.4% (Fig. 20a); after 30 minutes, the recovery increased to 28.7% (Fig. 20b). After 60 min of unloading, the second peak reached 23.7% of the first peak. Finally, after 200 min of unloading, the recovery was 23.1% of the peak crushing load (Fig. 20d).

**Figure 21:**
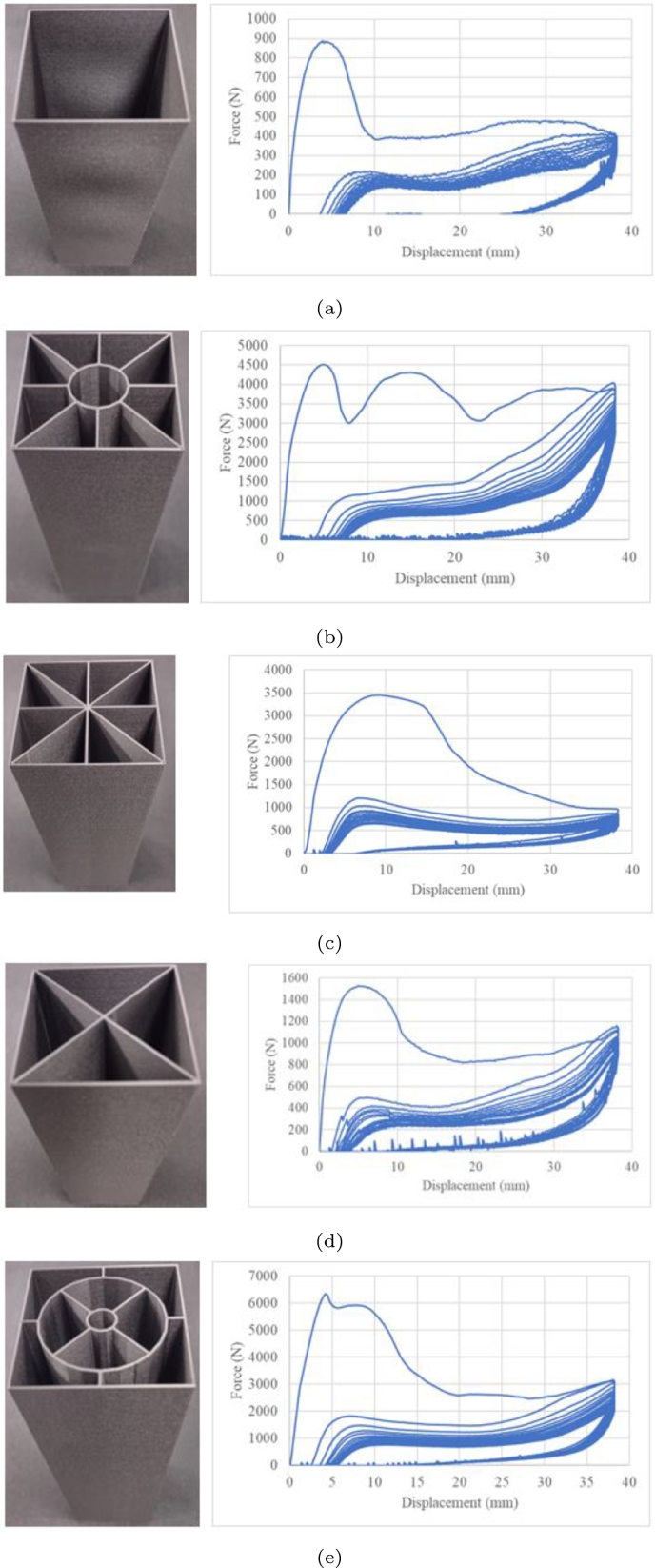
Low-cycle fatigue under crushing loads at 38.1 mm of displacement.

Five crash boxes were evaluated based on a displacement of 38.1 mm at a rate of 70 mm/min with an unload time of 30 min between load cycles (Fig. 21). The cyclic load was fifteen reversals. Although a recovery was observed for all cases, a permanent deformation was observed during unloading with a recovery of 10–23 mm. The recovery peak crushing loads are summarised in Table 2. The stiffness generated a stable unload behaviour (Figs. 21b–e). Fig. 21a shows a noisy unload response.

**Table 2:** Peak crushing force under cyclic crushing loads.

Cycle	Variant 1	Variant 2	Variant 3	Variant 4	Variant 5
1	885.3	4514.5	3445.5	1528.1	6340.1
2	243.5	1221.6	1204.3	499.3	1823.9
3	205	976.2	1037.7	421.8	1472.8
4	192.2	878.5	933.8	390.3	1282.9
5	191.3	794	917.8	386.5	1193.3
6	181.5	757.3	871.2	341.1	1089.3
7	174.9	719.6	830	327.8	1030.9
8	173.9	701.7	799.1	317.6	970.5
9	162.3	638.3	761	303.3	918.4
10	159.3	627.3	725.8	291.7	887.9
11	157.3	590.3	750	278.1	853.2
12	152.5	573	763.6	263.3	821
13	148.7	545.5	686	261.8	802.8
14	148	530.4	666	254.6	776.8
15	142.4	515.1	651.4	244.6	753.8

Table 2 shows the summary of the peak crushing loads in each cycle after monitoring subsequent cycles until 10 mm of displacement. A mean crush value was observed after this crushing displacement until it reached the load displacement ended. The absence of peaks suggested the generation of new folding, as observed for the first cycle load.

**Figure 22:**
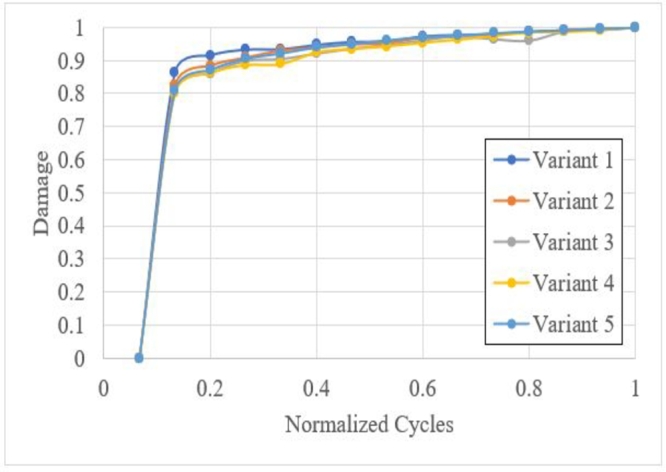
Accumulated damage under crushing loading.

Fig. 22 shows the damage response under various crushing loads. Variants 1, 2,3,4 and 5, correspond to Figs. 21a, b, c, d and e, respectively. All variants showed different evolution damage processes and minor damages than other variants. In some cycles, a lower damage was observed than the previous cycle, indicating component hardening.

**Figure 23:**
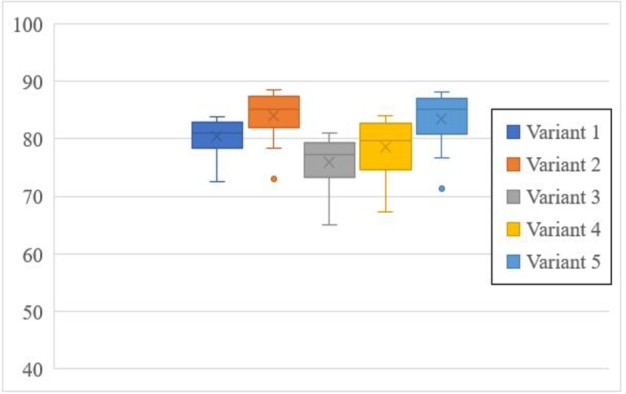
Damage dispersion.

The stiffness of the component is directly impacted by the *PCF* of current and subsequent cycles (Fig. 23). Variants 2,3 and 5 have a similar load dispersion; variant 4 shows major load dispersion, and variant 1 shows minor load dispersion with a noisy response (Fig. 21a).

**Figure 24:**
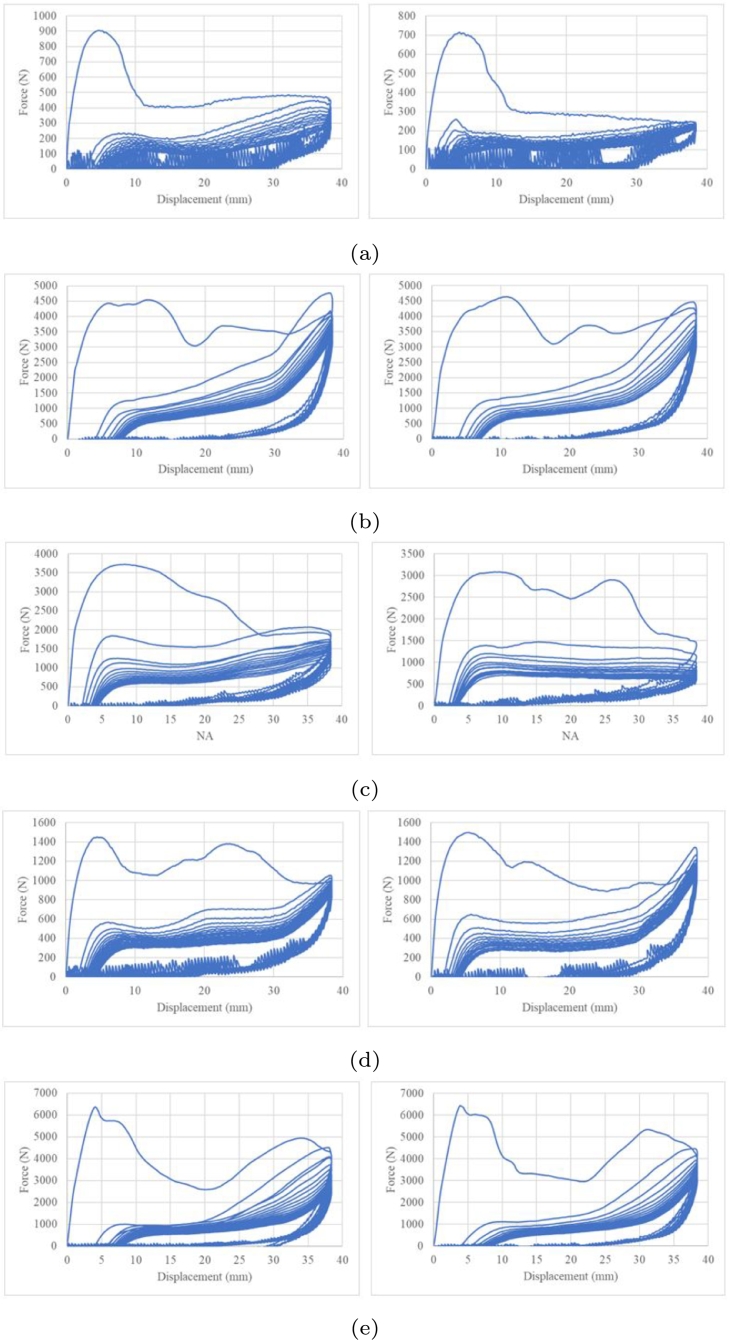
Reliability analysis second and third cyclic tests, (a) Variant 1, (b) Variant 2, (c) Variant 3, (d) Variant 4 and (e) Variant 5.

To evaluate the reliability of the energy absorber, two additional tests were performed per variant. The crushing force histories are shown in Fig. 24. The summary of the standard deviation of the three tests per variant is shown in Table 3.

**Table 3:** Standard deviation summary.

Cycle	Variant 1	Variant 2	Variant 3	Variant 4	Variant 5
1	0.0573	0.0121	0.0410	0.0118	0.0035
2	0.0229	0.0254	0.0934	0.0578	0.1369
3	0.0061	0.0310	0.0416	0.0466	0.1520
4	0.0184	0.0244	0.0461	0.0418	0.1236
5	0.0326	0.0302	0.0179	0.0282	0.1582
6	0.0278	0.0327	0.0182	0.0485	0.1609
7	0.0298	0.0326	0.0167	0.0475	0.1619
8	0.0398	0.0308	0.0264	0.0477	0.1417
9	0.0412	0.0583	0.0174	0.0529	0.2171
10	0.0443	0.0525	0.0243	0.0578	0.1667
11	0.0402	0.0514	0.0284	0.0617	0.1717
12	0.0414	0.0725	0.0363	0.0679	0.2017
13	0.0515	0.0938	0.0459	0.0627	0.2299
14	0.0729	0.1008	0.0314	0.0652	0.2391
15	0.0572	0.0892	0.0572	0.0706	0.2193
*μ*	0.0389	0.0492	0.0361	0.0513	0.1656

**Figure 25:**
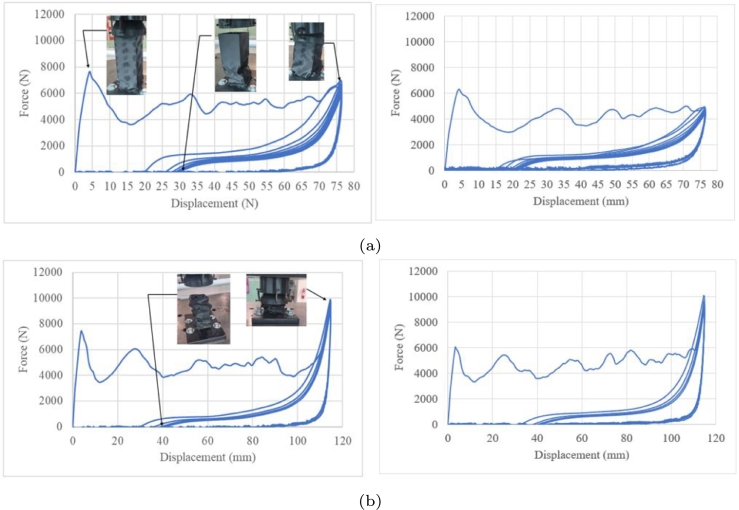
Low-cycle fatigue under crushing loads at (a) 76.2 mm and (b) 114.3 mm.

Fig. 25a shows the performance of the component until 76.2 mm of displacement and Fig. 25b shows the cyclic crushing load until 114.3 mm of displacement. These data were used to assess the performance of the component along the length.

During the first cycle, an interesting mechanical response was observed based on the *MCF*, although the recovery was not the same as that for the specimen with lower crushing displacements. The cyclic load test at 76.2 mm of crushing displacement was suspended at the ninth cycle because the peak crush load dropped at 38.1 mm below 10% of the $PCF_{o}$. At a crushing displacement of 114.3 mm, the failure criteria to stop the test was the same, i.e. *PCF* at 38.1 <10% of the PCF in cycle 1. The failure reached after only five cycles (Fig. 25a) and the recovery after unload was around 40 mm (Fig. 25b). The last peak was larger than the original due to it. The recovery was evaluated based on the major peak crush in every cycle at the end of the crushing load

The analysis of the viscoelastic behaviour of Onyx showed that it has recovery, which depends on the crushing load. Therefore, it can be used under low-cycle fatigue as a mechanical absorber. Moreover, the mean crush load can be managed using a crash box and similar peak crush tendency can be result in the end of displacement. Although at an initial stage of the displacement of the variant 5 the energy recovery has a detrimental behaviour with a marginal recovery, compared to the first peak load. It is observed that the energy dissipation capacity is increased in all load cycles at the end of the crushing displacement at 76.2 and 114.3 mm (Figs. 25a and 25b), which prevents an impact between critical components of the vehicle. This is based on a similarity of the non-linear stiffness behaviour of the stop springs, used in the suspension system, where at the end of their displacement their stiffness increases. To guarantee the repeatability of results, the tests were carried out twice at 76.2 mm and 114.3 mm, which correspond to 50% and 75% of the total length. When analyzing the dispersion of the peak crushing force of the four tests in Fig. 25, $s_{log}$=0.05 is obtained. When analyzing the dispersion by comparing the peak crushing force between both tests, an average dispersion $s_{log}$=0.034 and $s_{log}$=0.023 was obtained for the tests at 76.2 mm and 114.3 mm, respectively. Considering the peak crushing force at the maximum displacement, the mean dispersion is $s_{log}$=0.092 and $s_{log}$=0.002, for the 76.2 mm and 114.3 mm tests. Based on the requirement that the mean value of scatter allowed for uniaxial fatigue loads must be less than 0.3 [33], the values of all variants at 38.1 mm, 76.2 mm and 114.3 mm comply with the scatter requirement. These findings can be used to develop 3D printed components that can recover part of the adsorbed energy. These components are sustainable as they can be reused on the same vehicle until the damage reaches its critical value [34], [35].

Such printed components can also be used to improve the mechanical behaviour of crash boxes based on cyclic fatigue strength. Although this study focused on the impacts at low velocities in front end components, the results can be extended to applications of battery housings and other impact-absorbing devices such as sports helmets [36], [37], [38], [39]. Additionally, sensors can be installed in these component so that the drivers can be notified when a critical damage value is reached and suggest measures to replace the crash box. To ensure vehicle safety and prevent damage to critical vehicle components via instrumentation, a signal can be sent through an Internet of Things device to the vehicle supplier to contact the vehicle user and schedule the repair of the crash box [40].

## 7 Conclusions

Herein, the recovery of the peak crushing load under cyclic load and low-cycle fatigue by a 3D-printed material was evaluated. The component damage was evaluated based on the *PCF* during service life cycle assessment as a function of cycles to failure. This process allowed the measurement of damage, which was updated based on the direct and updated responses using non-destructive methods. The recovery time controlled by discharge cycle times was evaluated. Results showed that non-immediate impacts allowed an average recovery of 31.7% in the second cycle and 19.25% in the fifteenth cycle based on the first peak load. This prevented the underuse of the component when it could sustain a new load cycle. Thus, recovery and damage are related and depend on crushing displacement. Based on the analysis of experimental results the following conclusions can be drawn:

• Thin-wall components made of printed onyx, can be used as reusable crash box in low speed impacts.
• The recovery rate depended on the material geometry used for fabricating the crash box.
• Two stages of recovery were observed for printed Onyx during and after unloading. The cyclic behaviour caused hardening and accelerate the failure based on the component design and crushing
• Peak crushing loads at failure were defined based on vehicle characteristics, which depended on the loss of energy dissipation.
• The sustainable design of the proposed Onyx component extends the component life and reduces the carbon footprint.

## Statement

The authors did not receive support from any organization for the submitted work.

## CRediT authorship contribution statement

**Moises Jimenez-Martinez:** Writing – review & editing, Writing – original draft, Supervision, Methodology, Investigation, Formal analysis, Data curation, Conceptualization. **Guillermo Narvaez:** Visualization, Formal analysis. **Paulina Diaz-Montiel:** Writing – review & editing, Methodology.

## Declaration of Competing Interest

The authors declare that they have no known competing financial interests or personal relationships that could have appeared to influence the work reported in this paper.

## Data Availability

The authors confirm that the data supporting the findings of this study are available within the article.
